# Tumor Cell-Induced Platelet Aggregation as an Emerging Therapeutic Target for Cancer Therapy

**DOI:** 10.3389/fonc.2022.909767

**Published:** 2022-06-23

**Authors:** Wiktoria Strasenburg, Jakub Jóźwicki, Justyna Durślewicz, Błażej Kuffel, Martyna Parol Kulczyk, Adam Kowalewski, Dariusz Grzanka, Tomasz Drewa, Jan Adamowicz

**Affiliations:** ^1^ Department of Clinical Pathomorphology, Faculty of Medicine, Collegium Medicum in Bydgoszcz, Nicolaus Copernicus University, Toruń, Poland; ^2^ Department of General and Oncological Urology, Collegium Medicum in Bydgoszcz, Nicolaus Copernicus University in Toruń, Toruń, Poland

**Keywords:** TCIPA, platelets, aggregation, activation, cancer

## Abstract

Tumor cells have the ability to induce platelet activation and aggregation. This has been documented to be involved in tumor progression in several types of cancers, such as lung, colon, breast, pancreatic, ovarian, and brain. During the process, platelets protect circulating tumor cells from the deleterious effects of shear forces, shield tumor cells from the immune system, and provide growth factors, facilitating metastatic spread and tumor growth at the original site as well as at the site of metastasis. Herein, we present a wider view on the induction of platelet aggregation by specific factors primarily developed by cancer, including coagulation factors, adhesion receptors, growth factors, cysteine proteases, matrix metalloproteinases, glycoproteins, soluble mediators, and selectins. These factors may be presented on the surface of tumor cells as well as in their microenvironment, and some may trigger more than just one simple receptor–ligand mechanism. For a better understanding, we briefly discuss the physiological role of the factors in the platelet activation process, and subsequently, we provide scientific evidence and discuss their potential role in the progression of specific cancers. Targeting tumor cell-induced platelet aggregation (TCIPA) by antiplatelet drugs may open ways to develop new treatment modalities. On the one hand, it may affect patients’ prognosis by enhancing known therapies in advanced-stage tumors. On the other hand, the use of drugs that are mostly easily accessible and widely used in general practice may be an opportunity to propose an unparalleled antitumor prophylaxis. In this review, we present the recent discoveries of mechanisms by which cancer cells activate platelets, and discuss new platelet-targeted therapeutic strategies.

## Introduction

**Figure 1:**
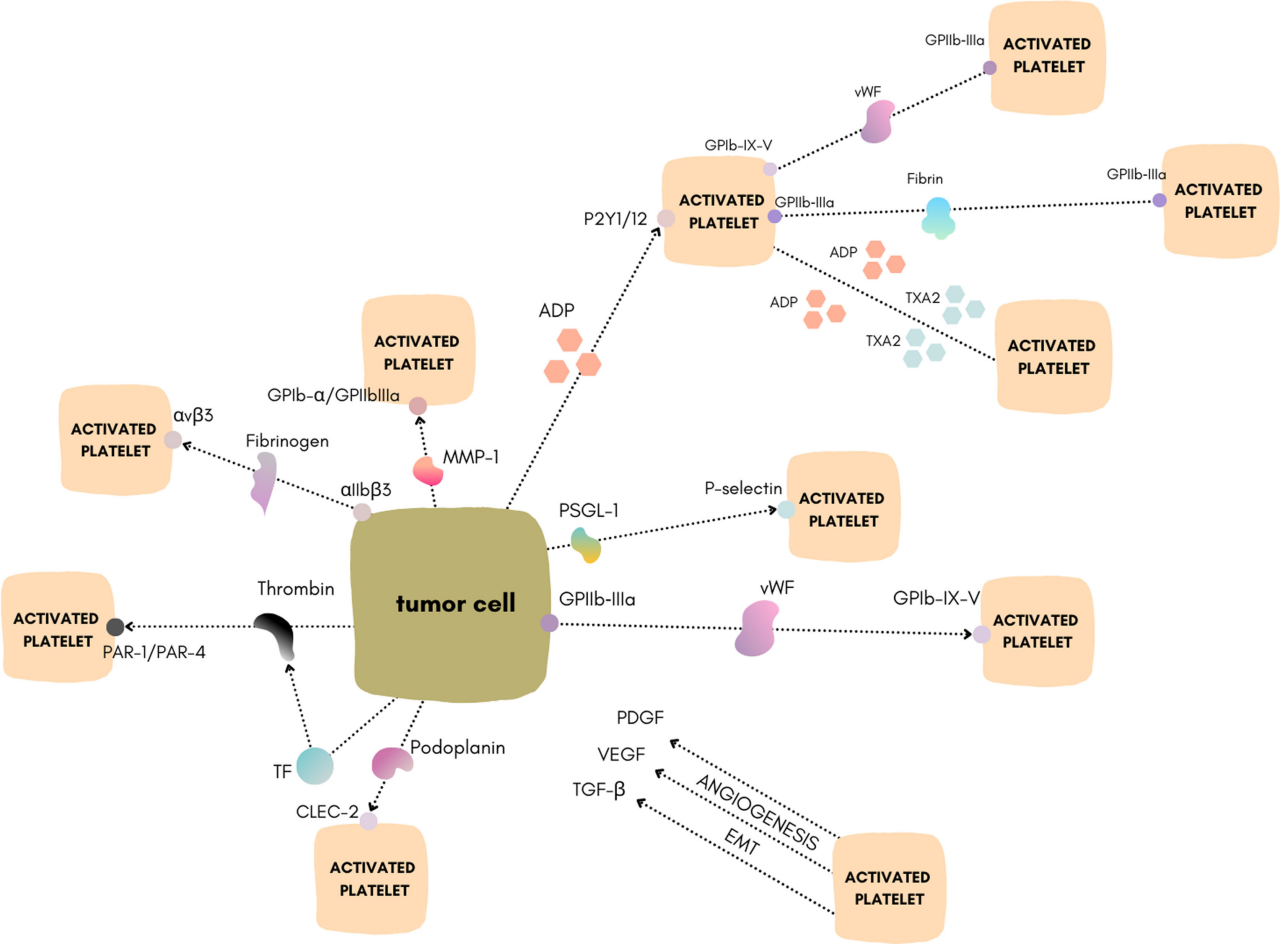
Schematic of the different mechanisms of TCIPA. Figure created using https://www.canva.com/.

The primary hemostatic function of platelets is well known; however, increasing evidence supports the crucial role of platelets in cancer biology (1, 2). In 1865 Armand Trousseau first described cases of thrombophlebitis in patients with cancer. He emphasized the association of malignancies with the creation of venous and arterial platelet–rich microthrombi in the vasculature (3). Further studies demonstrated that tumor cells can induce platelet activation and aggregation. This mechanism is now called tumor cell–induced platelet aggregation (TCIPA) (4). TCIPA has been documented to be involved in tumorigenesis in several types of cancers including breast (5)lung (6)and pancreatic (7). During TCIPAplatelets protect circulating tumor cells from the deleterious effects of shear forces and also preserve the tumor cells from the immune system by creating a physical barrier around cancer cells (8). These actions may contribute to metastatic spread and tumor growth (2). One of the reasons why TCIPA is in the spotlight of current research is the explored possibility to involve antiplatelet drugs in cancer therapy. Targeting TCIPA with antiplatelet drugs may open new ways to affect cancer environment and develop new treatment modalities. In this reviewwe present the recent discoveries of mechanisms explaining platelet activation by cancer cells. Moreoverwe discuss new platelet–targeted therapeutic strategies as potential inhibitors of TCIPA (**Figure 1**).

## Clotting Factors

### Thrombin

Thrombin is a serine proteaseplaying a pivotal role in blood coagulation (9). It converts fibrinogen into fibrin and activates various coagulation factorsincluding VVIIIXIand XIIIand the protease–activated receptors (PAR) on plateletsendothelial cellsmyocytesand neurons (2). In this processreceptorssuch as PAR–1PAR–3and PAR–4are activated (1). PAR–1 is the most effective receptor for thrombin (10). There is increasing evidence suggesting a crucial role of thrombin in cancer biology (11). Thrombin is proven to be generated by lung cancer cells (6). Thrombin–activated platelets express factors that facilitate contact with tumor cells andin turnenhance TCIPA through full activation of specific membrane receptors on platelets (12). Similarlyoverexpression of the PAR–1 receptor was associated with cancer progression and development. As demonstrated by Cisowski et al.silencing or pharmacologic blocking of PAR–1 results in a significant decrease in motility of the lung cancer cell lines A549 and HOP62 (13). PAR–4 is overexpressed in colorectal and prostate cancer and PAR–3 in kidney and liver cancer (14). Thrombin also increases the surface exposure of GPIIb–IIIa on platelets and tumor cellsthereby enhancing the interactions between tumor cells and platelets (15). Another potential mechanism linking thrombin with tumorigenesis is its major function during fibrinogen activation and conversion to fibrin. Thrombin may promote abnormal and upregulated fibrin deposition within the tumor matrix. Fibrin itself has also binding motifs for numerous integrins like GPIIb–IIIa and αVβ3making it capable of influencing numerous cell types including platelets and tumor cells (12). Interestinglythrombin may also contribute to breast cancer metastasis via other factorsunrelated to the TCIPA mechanism. It cleaves osteopontin (OPN) andthusincreases its biological activity. Schulze et al. reported that inhibition of thrombin in breast cancer cells overexpressing OPN leads to its more indolent behavior (16).

### Tissue Factor

Tissue factor (TF) is a membrane glycoprotein that is crucial to initiate the extrinsic coagulation cascade (17). Expression of TF has been detected in several types of cancersincluding breast cancer (18)prostate cancer (19)and lung cancer (20). TF is expressed on the cell membrane to activate the plasmatic coagulation cascade (21) that causes the generation of thrombinwhich in turn induces platelet activation (2). Furthermoreit has been discovered that TF plays an important role in tumor angiogenesis and progression as well as in metastasis (22). TF expression is under the control of E–cadherinPTENK–rasand p53. The activation of E–cadherin and K–ras or the loss–of–function of PTEN and p53 results in the implication of the mitogen–activated protein kinase (MAPK)/phosphoinositide–3 kinase (PI3K) signaling pathway and the subsequent increase of TF expression (23–25). High TF expression is correlated with the histological grade and poor prognosis in some tumor typesincluding non–small–cell lung carcinoma (26) and breast cancer (27). In bladder cancer patientshigh TF serum levels were previously shown to be associated with rapid disease progression (28). A study by John et al. has shown that despite the high expression of TF in bladder cancer cellsthe plasmatic coagulation was not induced. The authors explained this phenomenon by the comparably high levels of thrombomodulin that binds and inactivates thrombin on the cell surface (29). The TF–bearing extracellular vesicles (EVs) can be secreted by cancer cells andthusmay trigger TCIPA (30). For instanceSasano et al. demonstrated that TF–expressing EVs from ovarian cancer cells impact platelet aggregation and thrombosis (31). TF+ EVs from two human pancreatic adenocarcinoma cell lines affect resting platelets and activate them via thrombin generation (30). Geddings and colleagues presented that patients with advanced breast cancer had elevated levels of TF–bearing EVs compared with healthy controls (30).

### von Willebrand Factor

von Willebrand factor (vWF) is an adhesive and multimeric glycoprotein present in plasmasynthesized by endothelial cells and megakaryocytes. vWF has a central role in primary hemostasis where it mediates platelet adhesion to the exposed extracellular matrix at the site of vascular damage (32, 33). vWF promotes platelet accumulation in the classical first wave of hemostasis by binding the platelet glycoprotein Ib–IX–V (GPIb–IX–V) complex (32). Monomers of pro–vWF undergo dimerization in the endoplasmic reticulum through C–terminal disulfide bonds and then undergo multimerization in the Golgi apparatus through N–terminal disulfide bonds (34). The newly synthesized vWF multimers are stored in the Weibel–Palade bodies (WPB) of endothelial cells and in the α–granules of megakaryocytes and platelets. vWF occurs in a range of sizesreferred to as vWF multimersincluding ultra–largehighintermediateand low molecular weight forms. In addition to endothelial and platelet–derived vWFthere is also another pool of circulating large heterogeneous multimers composed of repeating monomeric unitsup to 40,000 kDa in lengththat are the most biologically active form of vWF. This pool is reported to be released upon endothelial cell activation in response to inflammatory and ischemic injuries (35) and in response to a variety of factors such as thrombinhistamineadenosine diphosphate (ADP)collagenand other immune or tumor cell–secreted factors (32). The circulating multimers act dynamicallydepending on shear conditions or the presence of vessel wall damage. In low–shear conditionsplatelet adhesion is not permissible (36). On the other handplatelet–derived vWF exists as a hyposialylated glycoformrendering it less susceptible to ADAMTS13–mediated proteolysis (37). Intriguinglytumors may also sequester circulating vWF from plasma into the tumor stroma. It is reported that the main role in this process is played by the collagen–binding motif within the A3 domain of vWF (38). Taking into consideration these argumentsthe endothelial cell–derived pool of vWF seems to be more relevant in investigating TCIPA. It is considered that vWF is one of the major platelet adhesion ligands that may also mediate cancer progression and metastasis (39). Upon tumor–induced endothelial cell activationthe vWF within WPB is secreted into the lumen of the blood vessel as well as basolaterally into the subendothelium (40). In the tumor microenvironmentit can contribute to increased angiogenesisblood vessel permeabilityand epithelial–mesenchymal transitioningwhich was reported in osteosarcoma cells (41). Intraluminal accumulation of the vWF can result in the deposition of platelet–rich thrombin within the vasculature and serve to increase the number of metastatic focias it was found in the murine melanoma cell lines Ret and B16F10 (42). Yang et al. reported that patients with late–stage gastric cancer had higher serum levels of vWFand suggested that the expression of vWF in gastric cancer cells may contribute to its progression in vivo. The authors also found that this may be regulated by the vascular endothelial growth factor (VEGF)–VEGFR2 signaling pathway (43). Endothelial cell activation followed by vWF fiber formation was found to be the main culprit of platelet aggregation in malignant melanoma vasculature (44). On the other handTerraube et al. found that the presence of vWF plays a protective role against murine melanoma and lung cancer metastasis in vivo (45). Studies reported that gastric cancer cells express vWFsecrete it into the circulationand thus mediate TCIPA. vWF potentiates TCIPAwhile inhibition of this factor reduces platelet–cancer cell interactions (46). vWF–cleaving protease (ADAMTS13) serum levels are associated with poor prognosis and metastasis (47, 48). Jurasz et al. have shown that vWF potentiates the platelet–aggregatory activity of human fibrosarcoma HT1080 cells. This effect appears to be mediated via upregulation of platelet GPIIb/IIIa (49). vWFin addition to promoting pro‐inflammatory signalingcan also regulate angiogenesis and vascular permeability andthereforecan facilitate tumor cell growth and extravasation (50).

## Adhesion Receptors

### Glycoprotein Ib–IX–V

GPIb–IX–V is a membrane receptor complex originating in megakaryocytes and belonging to the leucine–rich repeat family of proteins. The complex consists of four distinct transmembrane proteinsnamelyGPIbαGPIbβGPIXand GPVand is expressed on platelets. The most important component of the complex is the glycoprotein component GPIbα which contains the binding sites for vWFP–selectinthrombinthrombospondinfactor XIIfactor Xkininogenand integrin αMβ2 (Mac–1). GPIb–IX–V plays a critical role in thrombosisinflammationmetastasisand the life cycle of platelets (51–55). One of its functions is the interaction with vWF on sites of vascular injury. The GPIb–IX–V complex binds to vWF and initiates signaling that results in GPIIb–IIIa activation and platelet aggregation (56, 57). Several lines of evidence implicated the role of GPIb–IX–V in TCIPA. GPIb was recently discovered to be expressed in breast cancer cells (22, 58). It plays an important role in tumor cell–host cell interactions. Deregulated expression of GPIbα is associated with cell transformation and global genomic destabilization (59). Interestinglyinhibition of GPIb–IX–V or vWF function reduced platelet–cancer cell interactions suggesting that these receptors play a role in tumor–induced platelet aggregation (58). GPIb–IX–V has also been shown to contribute to tumor malignancy and metastasis in lung cancer (60).

### Glycoprotein IIb‐IIIa

Glycoprotein IIb‐IIIa (GPIIb‐IIIa) is an important platelet membrane receptor for fibrinogenfibronectinand vWF. It provides adhesive properties andhenceis necessary for platelet aggregation (58). The function of the GPIIb‐IIIa receptor in TCIPA has been established for decades (61). Platelets recruited in TCIPA can attach to the surface of tumor cells by a GPIIb–IIIa–fibrinogen bridge to secure tumor cells from immune surveillance (62). It also contributes to tumor progression and metastasis (63). Studies on breast cancer cell lines show that expression of the GPIIb–IIIa subunit occurs on the surface of MCF–7 cells and plays an important role in tumor metastasis (58). As demonstrated by Zhang et al.platelet GPIIb–IIIa is involved in the formation of the human melanoma A375 cell complex with platelets. Evidence showed that blocking the function of platelet GPIIa–IIIb by antagonists or antibodies could prevent hematogenous cancer metastasis (64). This mechanism was also observed in a study on lung and prostate cancer cases (64, 65). These results suggest that GPIIa–IIIb plays a key role in tumor progression and metastasismaking it an interesting target for anticancer therapy (64–66). Apart from therapyadvances in understanding the TCIPA mechanism may result in developing new highly specific diagnostic methods targeting cancer cells at the molecular level. Yap et al. introduced a specifically designed antibody that binds an activated form of the integrin receptor GPIIb–IIIa. The authors claimed that this method could be a new approach for enhancing ultrasound and PET imaging of tumors (67).

### Integrin αvβ3

Integrin αvβ3 is a transmembrane heterodimer which belongs to the family of cell adhesion receptors (68). Platelets express αvβ3 integrin at their surfacewhich binds several adhesive proteins including fibrinogenfibronectinvWFand vitronectin. It is possible that the role of αvβ3 is triggering platelet adhesion and aggregation at sites of vascular injury (69). Moreoverthe αvβ3 integrin is expressed in breast cancer cells and may influence TCIPA by binding tumor cells to platelets using plasma proteins such as fibrinogen (70). The platelet GPIIb–IIIa can link fibrin with tumor αvβ3 and mediate tumor cell–platelet aggregation (71). In malignant melanoma cellsαvβ3 mediates platelet aggregation cell arrest during flow (72). This interaction creates a physical shield around cancer cells by protecting them from the deleterious effects of shear forces and immune cells (71, 72). Interestinglythe combined blockade of platelet GPIIb–IIIa and tumor cell–expressed αvβ3 is more effective at inhibiting tumor growth when compared with the single blockade of integrin receptors (73). The role of αvβ3 in tumor biology is even more complex since it plays an important role in tumor angiogenesisprogressionand metastasis (73). In recent yearsmany studies have demonstrated that the increased expression of αvβ3 integrin is related to a metastatic phenotype in many types of cancers such as ovarian (74) prostate (75)and breast (76).

## Growth Factors

### Vascular Endothelial Growth Factor

VEGF is the basic regulator of vascular growth. The actions of VEGF include the regulation of proliferationmigrationand permeability of endothelial cells. VEGF increases the expression of adhesion molecules and coagulation factors. It is stored in high quantities in platelet alpha granules (77). Elevated serum VEGF levels in cancer patients correlate with a poorer prognosis (78, 78). The molecules accumulated in platelets entrapped within the tumor matrix are gradually released depending on the protease activity such as the matrix metalloproteinase (MMP) family (79, 80). Extracellular proteases act on VEGF in several ways: on the one handthey can release matrix–bound VEGFon the other handthey can suppress VEGF’s proangiogenic activity. As a result of platelet interaction with cancer cellsVEGF is released into the tumor microenvironment and stimulates neoangiogenesiswhich ultimately enhances tumor growth (77). The overexpression of VEGF is one of the main factors leading to the occurrence and progression of cancerincluding renal cancerbreast cancernon–small cell lung cancerand pancreatic cancer (81). Another source is cellular hypoxiaoccurring in tumors rapidly overgrowing their blood supply. It induces the production of VEGF through factor–1α. The released VEGF binds to the VEGFR of endothelial cellsfavoring the formation of tumor–associated microvesselsand thusincreases tumor oxygen deliverydecreases hypoxiaand contributes to its further growth through positive feedback (82).

### Transforming Growth Factor Beta

Platelets are a potent reservoir of transforming growth factor–beta (TGF–β) carrying a higher proportion of this molecule in the blood (up to 40%). Its release from TCIPA is widely described in the literature (83, 84). Howeverthere is no available research on the TGF–β mechanism primarily triggering TCIPAleaving a promising field for further studies. TGF–β is one of the most pleiotropic cytokines belonging to the transforming growth factor superfamily that includes three different mammalian isoforms (1–3) and many other signaling proteins. TGF–β proteins are produced by all white blood cell lineages. They are secreted in a latent form in which they bind with two other polypeptides: latent TGF–β–binding protein (LTBP) and latency–associated peptide (LAP). Thereforethe regulation of TGF–β levels is unique among other cytokines as they are not dependent on transcription factors but rather on proteases (such as plasmin)catalyzing the release of its active form (85). In this situationTGF–β may be upregulatedbypassing the transcription factors’ alterations. Activated TGF–β complexes form a serine/threonine kinase complex that binds to TGF–β receptors. These receptors are composed of both type 1 and type 2 receptor subunits. After binding of TGF–βthe type 2 receptor kinase phosphorylates and activates the type 1 receptor kinase that activates different downstream substrates and regulatory proteinsinducing the transcription of several target genes that promote differentiationchemotaxisproliferationand activation of immune cells (86). Recentlysome authors have focused on the role of TGF–β in the downstream activation of VEGF in tumorsthus leading to settling in an environment that is both nutritious through angiogenesis (87) and immunotolerant (88). Howeverthe initial clinical experience with drugs selectively targeting the tumor neovasculaturesuch as bevacizumabsunitiniband sorafenibhas been sobering: the major clinical responses to these drugs are rare and have minimal effects on overall survival after long–term follow–up (89–91). Some authors have explained that these effects were due to the promotion of compensatory angiogenic pathways (92) as well as to NK attenuation or activation of other intracellular pathways. Another mechanism of TGF–β–related tumorigenesis is through the promotion of epithelial–mesenchymal transition (EMT) (93).

### Platelet–Derived Growth Factor

Platelet–derived growth factor (PDGF) is a two–chain polypeptidewhich belongs to the family of growth factors (94) and is a potent cell–cycle regulator acting on multiple levels and affecting numerous tissues and structures. Originallythe PDGF was discovered in plateletshoweverPDGF and PDGF–like peptides have been recognized in various normal and malignant cellsencompassing the bone matrix and osteosarcoma cell (95, 96). The mechanism of action of PDGF is mediated by a specific membranous receptor—the platelet–derived growth factor receptor (PDGFR). The receptor belongs to a large family of tyrosine kinases regulating cellular function and proliferation. PDGF is stored in the α–granules of platelets (97). Platelet–tumor cell crosstalk in the tumor microenvironment leads to platelet activation and secretion of stored growth factors. Platelet aggregation induced through the thrombin pathway leads to the release of the whole content from α–granules of plateletsincluding PDGFTGF–βand VEGF (94, 98–100). In addition to its main role of inducing tumor growthit can also promote angiogenesis and neovascularization (101). On the other handPDGF stimulation triggers the repression of platelet aggregation (102). Niitsu et al. demonstrated that the human cell line of fibrosarcoma proliferated more rapidly in a medium containing platelet lysatewith PDGF alone substantially promoting growth activity (103). Tsuruo et al. proved that stimulation with PDGF can affect the growth of metastatic clones of mouse colon adenocarcinoma in a concentration–dependent way. They hypothesized that the PDGF pathway might be engaged in the promotion of metastasis. Accordinglymigrating tumor cells that get “arrested” in microvessels may attract platelet adherence and activationultimately leading to their aggregation and formation of a “safe cuff” (104). It was proven that PDGF can play an important role in the EMT of prostate cancer cells. Overexpression of PDGF–D (a variant of PDGF) in prostate cancer cells was related to enhanced adhesive and invasive behaviors and increased tumor growth (105). An additional example of the impact of PDGF on EMT was demonstrated in hepatocellular carcinoma and was linked with TGF–β–mediated progression (106). Activation of PDGFR with PDGF was proven to stimulate cellular proliferation in autocrine and paracrine ways (107). A couple of studies have shown that intraplatelet PDGF concentrations were significantly elevated in patients with colorectal cancer when compared to healthy individualswhich may indicate an even more important in–vivo interaction between platelet–produced PDGF and the tumor microenvironment (108109). Interestinglyit was shown that PDGF–producing platelets are expressing PDGFR on their own surface allowing autocrine feedback regulation of PDGF release. A study has shown the inhibitory influence of the activated PDGFR–alpha variant on platelet activation (108). The findings presented lead us to the hypothesis that PDGF plays a substantial role in tumor progression and metastasis.

## Cysteine Proteases

### Cathepsins

Cathepsins are a family of globular proteases that primarily were discovered as intracellularly functioning peptide hydrolaseshowevermultiple cathepsins have extracellular activity (109). The cathepsin family consists of a number of proteases named from “A” to “X” (110). Cathepsins play different physiological rolessuch as bone remodeling and activation of granzymes and mast cell proteases triggered by cathepsin K and Crespectively. In tumorscathepsins contribute to the maintenance of inflammatory processes. Neverthelesstheir main function is associated with tumor progression and metastasis. Cysteine cathepsins function in concert with serine proteases and matrix metalloproteinases (111). The expression of human cysteine cathepsins is highly upregulated in numerous cancerssuch as melanomacolorectal cancerglioblastomaprostate carcinomabreast carcinomalung cancerbladder cancerand gastric cancer (111–113). Cathepsin G seems to be involved in platelet activation in Trousseau syndrome (114): circulating mucins trigger granulocyte activationand in turngranulocytes release cathepsin Gwhich splits the PAR–4 and stimulates G proteins (Gq and G12/13) to prompt the shape change and activation of platelets (114, 115). Cathepsins K and B are secreted to an extracellular matrix as soluble enzymes where they remain in the active form (116–119). It has been found that overactivity of cathepsin K stimulates the initiation of the mTOR signal transduction pathway andthusthe proliferationmigrationand invasion of NSCLC cells (120). Under physiological conditionscathepsin B participates in the maintenance of cellular metabolism (106). Cathepsin B acts as a cysteine cathepsin often associated with tumor progression (121). Overexpressed cathepsin B level was associated with the notably shorter overall survival of colon cancer patients (122). A strong correlation between cathepsin B expression and tumor angiogenesisinvasionand metastasis has been widely described in the literature (123). Cathepsin B and cancer procoagulant factor (9) were widely described to participate in TCIPA (124–126).

## Matrix Metalloproteinases

MMPs are structurally similarzinc–dependent endopeptidases. The major function of the MMP family is controlled degeneration of the extracellular matrix (127). Their influence extends from embryonic developmentmorphogenesisand tissue remodeling to the regulation of vascular reactions and leukocyte and platelet activity (128). MMPs are involved in all steps of cancer progression: from primary tumor development to distant metastasis (129). The expression of MMP–2 on the surface of cancer cells was described in studies conducted on fibrosarcoma and colorectal and breast cancerwhere the authors have revealed that platelet and MMP–2 manifested by cancer cells contribute to TCIPA (130). Other MMPs of similar function include the membrane type I–matrix metalloproteinase (MT1–MMP)MMP–1and MMP–9also involved in platelet aggregation and TCIPA (131–134). MMP–1 expressed on breast cancer cells interacts with both GPIb–α and GPIIb–IIIaleading to their upregulation and providing ADP release and thus promoting TCIPA (2). The role of MMP–2 in the TCIPA pathway has been confirmed in HT1080 human fibrosarcoma cells and MCF–7 breast carcinoma cells (49, 134) as well as in human prostate cancer (130). The effect of MMP–2–induced platelet aggregation depends on the activation of proMMP–2 to MMP–2 through MMP–14 (126). It is considered that the communication between MMPs and glycoprotein receptors such as GPIIb–IIIaGPIband integrin αvβ3 is responsible for the MMP–mediated stimulation of platelets and tumor cells (135–137)howeverthe mechanism of action is still not completely understood (134). MMPs have presented the ability to stimulate TCIPA in vitrowhich is similar to cathepsin B (133). MMPs can be released from both platelets and tumor cells in vivo (138).

## Sialomucin Glycoproteins

### Podoplanin

Podoplanin (PDPN) is a mucin–type protein that mediates effects on cell migration and adhesion through its multiple partners. During embryonic developmentit plays a role in blood and lymphatic vessel separation by binding platelet C–type lectin–like receptor 1B (CLEC1B)triggering CLEC1B activation in platelets and leading to platelet activation and/or aggregation (139, 140). PDPN directly interacts with the CLEC2which promotes platelet aggregation and activation. Although elevated levels of PDPN have been correlated with increased malignancy in different tumorsits relevance for tumor progression is still unclear. Recentlya hypothesis of two different mechanisms of PDPN–related TCIPA in brain tumor patients has emerged:

1. PDPN may be released into the circulationeither in soluble form or on the surface of tumor–derived microvesicles.
2. Circulating tumor cells may be a source of circulating PDPN. Trapping of tumor cells in the venous system might lead to further local platelet activation and aggregation (141).

PDPN is known to contribute to tumor progression by inducing cancer cell migration and tumor invasion connected to the EMT mechanism (145146) and in the absence of EMT markers (142). High PDPN expression in primary brain tumors is associated with an increased risk of venous thromboembolism (VTE) (141) cancer progressionand overall poor prognosis (143, 144). Thereforethe PDPN–CLEC2 axis is a potential drug target for both reducing the risk of VTE and improving prognosis (141). Anti–PDPN therapies are expected to be of robust potential for future treatment strategies (145). In recent preclinical studiesa few anti–PDPN factors were targetedincluding types of recombinant immunotoxin NZ (146–148) and CD9an inhibitor of Aggrus/PDPN–induced platelet aggregation recognized to reduce the metastatic potential of HT1080 cells (149).

## Soluble Mediators

### Adenosine Diphosphate

ADP is a strong proaggregatory factoraccumulated in platelet–dense granulesand constitutes a secondary mediator of platelet aggregation (124). ADP has the capacity to communicate with platelet receptors P2Y12 and P2Y1resulting in the activation of platelet aggregation and changes of shape as well as the release of thromboxane A2 (TXA2) by platelets (150) and other multiple growth factors (151–153). ADP has been found to be expressed on cancer cells and involved in TCIPA. The P2Y12 receptor plays the main role in the process (134, 154). ADP–induced platelet activation is connected normally to VEGF release. Bambace et al. proved this by the termination of platelet activation by selective repression of the P2Y12 receptor (155). Interestinglyderegulation of ADP molecules may influence the reduction of metastases. Uluçkan et al. examined the mouse models of breast cancer and melanoma metastases treating them with acetylsalicylic acid and APT102 (a soluble apyrase/ADPase). Their results stand in favor of anti–ADP therapy in cancer (156).

### Thromboxane A2

TXA2 is considered a powerful modulator of platelet activation and aggregation as well as a stimulator of vascular constrictionwhich acts via binding to the thromboxane prostanoid receptor (TP) (157). TXA2 is considered a crucial molecule associated with tumor metastasis. Some evidence supported this hypothesis: 1) TXA2 is a strong platelet–aggregatory eicosanoidfacilitating the binding of tumor cell–platelet aggregates to the surface of endothelial cells (158). 2) TXA2 enhanced the migration and angiogenesis of endothelial cells in both in–vitro and in–vivo models (164165). FurthermoreTXA2 and ADP are recognized as “soluble stimulators” of platelet aggregation (159–161). TXA2similar to ADPis secreted in an autocrine/paracrine manner and triggers platelet activation through positive feedback (162). The release of both TXA2 and ADP factors stimulates the conversion of the GPIIb/IIIa receptor into an active form mediating platelet aggregation. Lian et al. have noticed that both TXA2 and ADP signaling pathways are prompted during the MCF–7 cell–initiated TCIPA (162). TXA2 synthesis is catalyzed by cyclooxygenase 1 (COX–1). COX–1 in platelets enzymatically converts arachidonic acid into PGG2 and then into PGH2 and generates prothrombotic TXA2. Lucotti and colleagues have provided evidence that aspirin reduces the metastasis of different murine tumors (melanomabreast cancercolorectal cancer) by inhibition of platelet COX–1 and its product TXA2. High and medium doses of aspirin reduced the number of metastatic lung nodules by more than 50%. Howeverthe authors concluded that the use of more specific TXA2 inhibitorssuch as picotamidecould be more beneficial since they do not affect gastroprotective COX–1 products (163). Recent meta–analyses of 88 cohort trials have revealed that routine aspirin administration correlates with diminished risk of several types of cancersincluding colorectalgastricbreastand prostate. Unexpectedlythere was no correlation with the risk of lung cancer (164).

## Selectins

### P–selectin

P–selectin is a Ca2+–dependent receptor for myeloid cells that binds to carbohydrates on neutrophils and monocytes (165). It mediates the interaction of activated endothelial cells or platelets with leukocytes. The ligands recognized are sialyl–Lewis X (sLeX) and P–selectin glycoprotein ligand 1 (PSGL–1) (166). P–selectin functions as a cell adhesion molecule (CAM) on the surfaces of activated endothelial cells and activated platelets. In inactive endothelium and plateletsit is stored within the Weibel–Palade bodies and α–granulesrespectively. It is responsible for rapid leukocyte rolling over vascular surfaces during the initial steps of inflammation. It is widely known that P–selectin induces TCIPA andthuspromotes tumor growth (167). The rapid mobilization of P–selectin primarily to TCIPA was observed in tumor blood vessels in different speciessuch as oil miceC57BL6 miceand nude miceand in different tumor types like lung carcinomacolon carcinomabreast carcinomaand gliomas in response to radiotherapy. In contrastnormal tissue did not reveal increased post–treatment expression (168). This may suggest that the tumor and its environment can stimulate P–selectin to be in a closely preactivated stateready to externalize when a non–specific trigger occurs (such as radiation therapy). Other authors noticed P–selectin upregulation to be triggered by contact with the tumor cell–surface mucin (169) and non–mucin ligands (170). Mucins associated with cancer progression are MUC1MUC2MUC4and MUC16. As demonstrated by Kim et al.large mucin molecules on the surface of tumor cells bearing multiple P–selectin–binding sites could bridge tumor cells and P–selectin–expressing platelets (167). These interactions protect tumor cells within the bloodstreamhiding them from NK cells (1)which could influence metastatic spread and may also contribute to tumor progression (171). Studies on mice show that platelet–tumor cell interactions are significantly reduced in P–selectin–deficient miceand consequentlyattenuation of metastasis is observed. Furthermoreenzymatic removal of carcinoma mucins results in attenuated metastasis comparable to the absence of P–selectin (172).

An abbreviated description of all aforementioned factors is gathered in **Supplementary Table 1**.

## TCIPA Targeting in Cancer Management

In recent yearsthe successful adoption and implementation of selective cancer therapies in clinical practice has increased research efforts aimed to identify and target various anticancer mechanisms. TCIPA is one of the pathways explored in the search for new options for cancer treatment. The crosstalk between plateletstheir receptorsreleased moleculesand clotting factors is subject to extensive and long–time research from different medical fields. These extensive studies resulted in the development of blockbuster therapies in cardiology and vascular medicine. The major principle of those protocols is to affect aggregation and clotting in a safe and controlled manner on different regulatory levels. The rich experience gained in the design of antiplatelet and antithrombotic treatment modalities may be transferred to oncology. For instanceTCIPA is a mechanism worthy of further studies as it demonstrates the involvement of platelets in carcinogenesis. It is highly probable that TCIPA inhibition would be beneficial for patients due to reduced risk of cancer–related thrombosis and associated clinical conditions such as strokepulmonary embolismand deep vein thrombosis. Howeverin this articlewe focused on exposing antitumor effects mediated by influencing TCIPA. The proof of concept is several studies reporting the potential benefit of targeting TCIPA in various tumors.

### Thrombin and Factor X

In pancreatic cancerthe expression of the PAR–1 receptor in the tumor microenvironment was proven to drive progression and induce chemoresistance (173). Thereforethe next step was to use a thrombin inhibitor in cancer therapy. In a study on micea direct thrombin inhibitor dabigatranwidely used as an anticoagulant drug in numerous indicationswas employed. The study showed that it significantly potentiated gemcitabine–induced growth inhibition of pancreatic cancer (174).

Dabigatran and a direct factor Xa inhibitor rivaroxaban are known as novel oral anticoagulants (NOACs) and have gained widespread use in medicine. The latter was found to inhibit cancer stem cell (CSC) activity in the in–vitro functional CSC assay of mammosphere formation (175). Thenin 2020it was included in a clinical trial to evaluate the impact of rivaroxaban on tumor progression in ER–negative stage I–III early breast cancer patients. Up to datethe results of the trial are still concealed. Another indirect antitumor mechanism of antithrombotic drugs is the reduction of angiogenic potential by limiting the VEGF platelet release (176).

### Adhesion Receptors

The inhibition of adhesion receptors using monoclonal antibodies has been already the subject of several studies. Qi et al. revealed that inhibition of GPIba leads to reduced interaction between platelets and tumor cellswhich results in the diminished metastatic potential of lung cancer cells. The study was performed in vitro and in vivo on animal models (177). Another study by Zhang et al. has demonstrated the promising effect of the anti–GPIIIa antibody on lung carcinoma cells in rat models. The mechanism of action was based on the fragmentation of activated platelets (178). The inhibition of GPIIb/IIIa in breast cancer cells was studied by Kononczuk et al. They have used specific antagonists of GPIIb/IIIa—abciximab and eptifibatide—to observe their proapoptotic effect on human breast cancer cells (179). Their promising results encouraged further trials. Another experimental anti–GPIIb/IIIa antagonist is the newly synthesized XV454 tested for its anticancer properties against lung cancer in rats. The results indicated a significant influence of this drug on tumor cell–platelet interaction and metastasis (65).

### PDPN and CLEC2

The PDPN–CLEC2 axis might provide a potential drug target for both reducing the risk of VTE and improving prognosis. Anti–PDPN therapies are expected to be of robust potential for future treatment strategies. In recent preclinical studiesseveral molecules were evaluated to interfere with the PDPN–CLEC2 pathway. CD9an inhibitor of PDPN–induced platelet aggregationwas recognized to reduce the metastatic potential of human fibrosarcoma cells (149). The recombinant immunotoxin NZ–1–(scdsFv)–PE38KDEL was recognized to delay the growth of glioblastoma and medulloblastoma tumor cells (144). The first synthesized selective inhibitor of PDPN–CLEC2 interaction is the 5–nitrobenzoate compound 2CP. Chang et al. proved its selective TCIPA inhibition on osteosarcoma and glioma cells and cisplatin therapy efficacy augmentation (180).

### Soluble Mediators

Another potential target is the ADP receptor P2Y12. The study of Cho et al. has shown that the growth of ovarian cancer in murine models was reduced with the specific P2Y12 inhibitor ticagrelor (181). Aspirin’s effect on platelet–mediated tumor progression is another potential therapeutic target: the study of Guillem–Llobat et al. has proven that COX–1 inhibition by aspirin could lower the metastatic potential of human colon adenocarcinoma cells (182). Ifetrobana potent selective TXA receptor antagonistpresumed to be decreasing cancer metastatic potentialhas been recently included in a second phase clinical trial involving patients with malignant solid tumors at high risk of metastatic recurrence. The results of that trial are planned to be available after 2025 (183).

### P–selectin

Studies reported that heparin is an outstanding inhibitor of P–selectinwhich binds to its natural ligandsthus inhibiting the initial platelet–tumor cell interactions. Even a single heparin dose that transiently blocks this interaction is sufficient to prevent long–term organ colonization. These discoveries indicate that P–selectin and its ligands could be a potential therapeutic target (169).

The therapeutic targets among the TCIPA mechanisms are shown in **Supplementary Table 2**.

## Conclusion and Future Perspectives

Among the unorthodox mechanisms of tumor progressionTCIPA has established by far a genuine target for potential therapies. The discussed studies demonstrated the encouraging influence of newly manufactured as well as widely used antiplatelet drugs on the inhibition of tumorigenesisprogressionand metastasis. These revelations should lay the groundwork for next–level clinical trials to optimize and determine the oncological efficiency of antiplatelet treatment. Howeverbecause of the simultaneous influence on blood coagulation and the wide variety of individual sensitivity to antiplatelet drugsit is still difficult to specify the optimal criteria for such studies. Hopefullyfurther research in the field of TCIPA will soon give us another instrument for aiding antitumor therapies.

## Author Contributions

The first draft of the manuscript was written by WS, JJ, JD, BK, MK, and AK. Supervision and review were exercised by DG, TD, and JA. All authors contributed to the article and approved the submitted version.

## Funding

This research was funded by Nicolaus Copernicus University in ToruńFaculty of MedicineCollegium Medicum in Bydgoszcz (Research Task No. 141 within the framework of the Basic Research Activity).

## Conflict of Interest

The authors declare that the research was conducted in the absence of any commercial or financial relationships that could be construed as a potential conflict of interest.

## Publisher’s Note

All claims expressed in this article are solely those of the authors and do not necessarily represent those of their affiliated organizations, or those of the publisher, the editors and the reviewers. Any product that may be evaluated in this article, or claim that may be made by its manufacturer, is not guaranteed or endorsed by the publisher.
