# Supplementary material for: Tumor Cell-Induced Platelet Aggregation as an Emerging Therapeutic Target for Cancer Therapy
Source: Front Oncol. 2022 Jun 23;12:909767. doi: 10.3389/fonc.2022.909767 (PMC9259835; doi:10.3389/fonc.2022.909767)
Supplement: Supplementary file 1 [file Table_1.docx]

Supplementary Material

| **Category** | **Agents** | **Role in TCIPA** |
| --- | --- | --- |
| **Clotting factors** | Thrombin | - Activates PAR1 and PAR4 receptors on platelets and leads to platelet activation and aggregation. - Converts soluble fibrinogen in insoluble fibrin due to clot formation and retraction. - Activates factors V, VIII, XI and XII to amplify coagulation. |
|  | TF | - Forms active TF/VIIa complex to proteolytic activation of factors IX and X, where stimulated factor Xa leads to conversion of pro-thrombin to thrombin, thus induces platelet activation. |
|  | vWF | - Binds several ligands such as GPIb, aIIbb3 or aVb3 simultaneously and directly interacts with tumor cells. - Increases the adhesion of tumor cells to endothelial cells. |
| **Adhesion receptors** | GPIb-IX-V | - GPIbα subunit of the GPIb-IX-V complex contains the binding sites for several molecules, including vWF, P-selectin (CD62P), thrombin thrombospondin-1, coagulation factors XI and XII, high molecular kininogen and integrin Mac-1 (CD11b/CD18). |
|  | GPIIb-IIIa | - Binds to fibrinogen, von Willebrand factor, fibronectin and vitronectin. - GPIIb-IIIa-fibrinogen bridge is responsible for the formation of stable platelet aggregates. |
|  | Integrin αVβ3 | - αvβ3 supports modest platelet adhesion to both fibronectin and vitronectin and might participate in clot retraction. |
| **Growth factors** | VEGF | - Tumor cell-derived ADP induces release of VEGF through stimulation of P2Y12 receptor, favouring the formation of microvessels and resulting even more tumor vascularization. |
|  | TGF-β | - The activated alpha (α) granules, located in platelets (through the direct contact with the malignant cells) secrete TGF-β, which induces tumor growth, promotes angiogenesis and tumoral neovascularization. |
|  | PDGF | - The activated alpha (α) granules, located in platelets (through the direct contact with the malignant cells) secrete PDGF, which induces tumor growth, promotes angiogenesis and tumoral neovascularization. |
| **Cysteine proteases** | Cathepsins | - Cathepsin B induce aggregation when released from cancer cells an effect that may be related to the generation of oxygen-derived free radicals by platelets. - Cathepsin G is involved in indirect mechanism of platelet activation through the tumor cells. In a way of bidirectional signaling and release of Cat G by the granulocytes it comes to cleave the platelet protease activated receptor-4 (PAR4) and activate G proteins (Gq and G12/13) to change the shape and activate platelets. - Cathepsin K is responsible for platelet aggregation in a way of dose-dependend manner by proteolytically-activated receptors PAR 3 and 4. |
| **Matrix metallopro-teinases** | MMPs | - Interact with glycoprotein receptors, leading to their upregulation as well as release of ADP and thromboxane which activate TCIPA. - The MT1-MMP-MMP-2 complex may lead to activation of platelet GPIIb/IIIa during TCIPA. |
| **Sialomucins glycopro-teins** | PDPN | - Directly binds the platelet receptor C-type lectin-like receptor (CLEC-2) and induces platelet activation and aggregation |
| **Soluble stimulators** | ADP | - Communicates with two major ADP-specific G-coupled receptors (P2Y12 and P2Y1) on platelets, resulting in platelet shape changes, activation of platelet and release of TXA2 by platelets   and platelet aggregation |

**
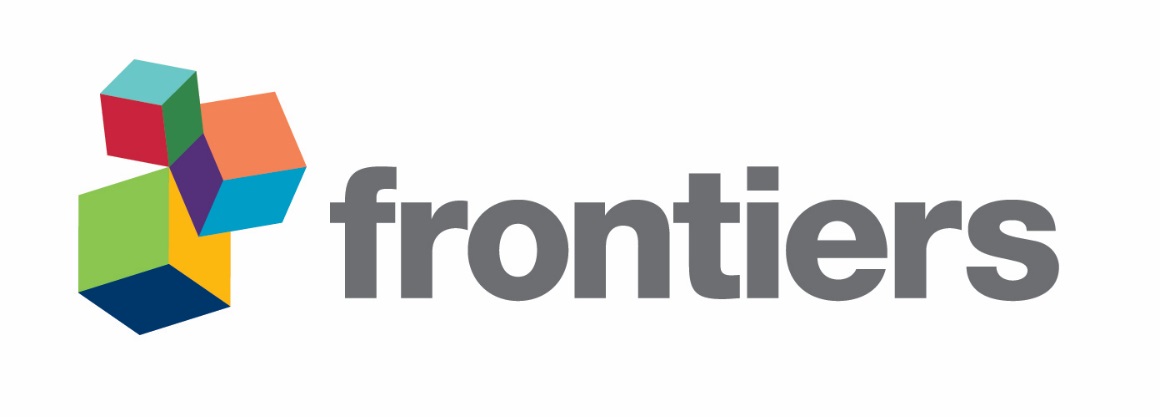
**

**Supplementary Tables1.**Agents involved in tumor cell induced platelet aggregation (TCIPA).
