# Supplementary material for: Tumor Cell-Induced Platelet Aggregation as an Emerging Therapeutic Target for Cancer Therapy
Source: Front Oncol. 2022 Jun 23;12:909767. doi: 10.3389/fonc.2022.909767 (PMC9259835; doi:10.3389/fonc.2022.909767)
Supplement: Supplementary file 2 [file Table_2.docx]

Supplementary Material

| **Tumor** | **Target** | **Drugname** | **Mechanism** | **Clinical trial** | **Author** |
| --- | --- | --- | --- | --- | --- |
| **Pancreatic cancer** | Thrombin&Factor X | Dabigatran | Anti-thrombin |  | Shi K. et al. |
| **Breast cancer** | Thrombin&Factor X | Rivaroxaban | Anti-FXa | + | Castle J. et al. |
|  | Adhesionreceptors | Abciximab,  Eptifibatide | Anti-GPIIb/IIIa |  | Kononczuk J. et al. |
| **Lung cancer** | Adhesionreceptors | mAb1D12 *  mAb2B4 | Anti-GpIba |  | Qi Y. et al. |
|  |  | A11** | Anti-GpIIIa |  | Zhang W. et al. |
|  |  | XV454*** | Anti-GPIIb/IIIa |  | Amirkhosravi A. et al. |
| **Colon cancer** | COX | Aspirin | Anti-COX1 |  | Guillem-Llobat P. et al. |
|  | P-selectin | Heparin | Blocking P-selectin-basedplateletinteractions |  | Borsig L. et al. |
| **Ovarian cancer** | ADP | Ticagrelor | P2Y12-receptor antagonist |  | Min SoonCho et al. |
| **Glioblastoma and medulloblastoma** | PDPN& CLEC | NZ-1**** | Anti-PDPN |  | Chandramohan V. et al. |
| **Glioma and osteosarcoma** |  | 2CP***** | Anti-PDPL-CLEC2 |  | Chang Y-W. et al. |
| **Fibrosarcoma** | PDPN& CLEC | CD9 | Anti-PDPN |  | Nakazawa Y. et al. |
| **Various solid cancers** | COX | Ifetroban | Anti-TXA | + | Mayer I. |

*mAb – monoclonalAntibody,**single-chainantibody (scFv Ab) againstintegrin GPIIIa49-66 (named A11),***novel non-peptideGpIIb/IIIaantagonist XV454,**** NZ-1-(scdsFv)-PE38KDEL,*****5-nitrobenzoate compound 2CP.

**
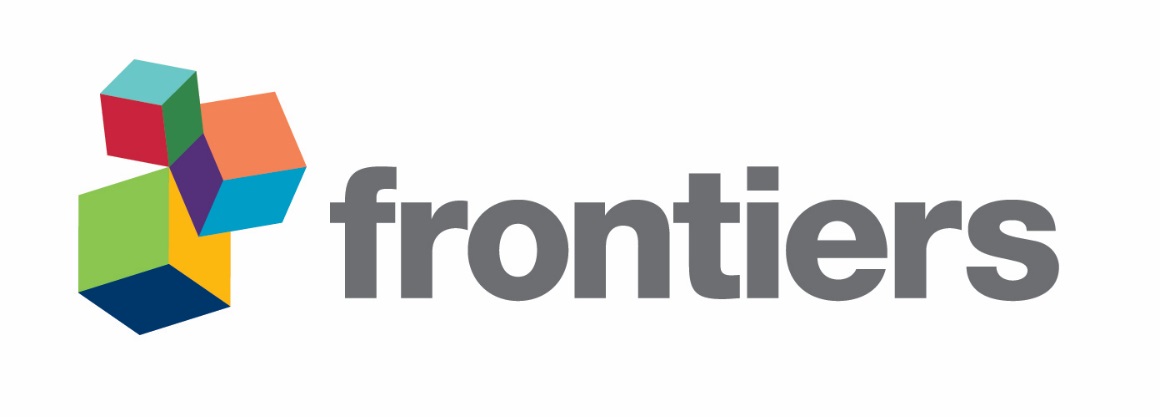
**

**Supplementary Table 2.** Recent therapeutic targets among TCIPA mechanisms with anti-tumor potential.
